# Nodulation and Growth Promotion of Chickpea by *Mesorhizobium* Isolates from Diverse Sources

**DOI:** 10.3390/microorganisms10122467

**Published:** 2022-12-14

**Authors:** Edwin I. Wanjofu, Stephanus N. Venter, Chrizelle W. Beukes, Emma T. Steenkamp, Eastonce T. Gwata, Esther K. Muema

**Affiliations:** 1 Department of Biochemistry, Genetics and Microbiology, Forestry and Agricultural Biotechnology Institute (FABI), University of Pretoria, Pretoria 0002, South Africa; 2 The James Hutton Institute, Invergowrie, Dundee DD2 5DA, UK; 3 Department of Plant and Soil Sciences, Faculty of Science, Engineering and Agriculture, University of Venda, Thohoyandou 0950, South Africa

**Keywords:** chickpea, *Mesorhizobium*, nodulation, symbiotic effectiveness, naturalized strains

## Abstract

The cultivation of chickpea (*Cicer arietinum* L.) in South Africa is dependent on the application of suitable *Mesorhizobium* inoculants. Therefore, we evaluated the symbiotic effectiveness of several *Mesorhizobium* strains with different chickpea genotypes under controlled conditions. The tested parameters included shoot dry weight (SDW), nodule fresh weight (NFW), plant height, relative symbiotic effectiveness (RSE) on the plant as well as indole acetic acid (IAA) production and phosphate solubilization on the rhizobia. Twenty-one *Mesorhizobium* strains and six desi chickpea genotypes were laid out in a completely randomized design (CRD) with three replicates in a glasshouse pot experiment. The factors, chickpea genotype and *Mesorhizobium* strain, had significant effects on the measured parameters (*p* < 0.001) but lacked significant interactions based on the analysis of variance (ANOVA). The light variety desi genotype outperformed the other chickpea genotypes on all tested parameters. In general, inoculation with strains LMG15046, CC1192, XAP4, XAP10, and LMG14989 performed best for all the tested parameters. All the strains were able to produce IAA and solubilize phosphate except the South African field isolates, which could not solubilize phosphate. Taken together, inoculation with compatible *Mesorhizobium* promoted chickpea growth. This is the first study to report on chickpea-compatible *Mesorhizobium* strains isolated from uninoculated South African soils with no history of chickpea production; although, their plant growth promotion ability was poorer compared to some of the globally sourced strains. Since this study was conducted under controlled conditions, we recommend field studies to assess the performance of the five highlighted strains under environmental conditions in South Africa.

## 1. Introduction

Nitrogen comprises an essential and large percentage of all living organisms’ biomolecules [1]. However, its bioavailability is often limited, despite the element’s high abundance on Earth [1]. This is because atmospheric nitrogen is inert and only becomes available for biological processes in reduced or fixed forms such as NH_4_^+^ and NO_3_^−^ [2,3]. These compounds are essential for soil health [1], and in many agricultural settings, nitrogen availability is routinely augmented by the addition of chemically synthesized fertilizer [4,5,6]. Although this is a practical solution, it is expensive and has long-lasting, far-reaching detrimental effects on the environment [7].

Biological nitrogen fixation is an environmentally friendly alternative to overcome nitrogen insufficiency in poor soils [4,5,6,8]. Globally, this process accounts for the fixation of approximately 50–70 Tg of N year^−1^ [9,10]. It involves the conversion of atmospheric nitrogen to bioavailable forms by certain prokaryotes [11]. These nitrogen-fixing microorganisms, collectively called diazotrophs, are either free-living or occur as symbionts of certain plants [12]. Among the best-studied examples are symbiotic bacteria that form a mutualistic relationship with legumes, which result in the formation of root or stem nodules within which the bacteria fix nitrogen for their host while receiving fixed carbon from photosynthates [13,14]. These symbiotic bacteria are referred to as rhizobia and have various members in various genera of the *Alpha*- and *Betaproteobacteria* [15,16,17]. 

Specificity in the legume-rhizobium symbiosis depends largely upon signal exchange between the partners. This is dictated by both the ability of the rhizobia to perceive plant exudates and the plant’s reception of the rhizobial Nod Factors [18,19]. More promiscuous legumes recognize a wider range of Nod Factors, with notable examples being cowpea (*Vigna unguiculata* (L.) Walp.) and siratro (*Macroptilium atropurpureum* (DC.) Urb.) that nodulate with rhizobia from diverse species harboring different sets of nodulation (*nod*) genes [18,20,21,22]. Other legumes may be more restrictive or specific [18,19]. Chickpea (*Cicer arietinum* L.), for example, is primarily nodulated by strains of *Mesorhizobium ciceri* or *Mesorhizobium mediterraneum* [18,20,21,22], harboring a very conserved set of *nod* genes [23]. Although a few other *Mesorhizobium* species are capable of chickpea nodulation [24,25,26], it has been proposed that they obtained the required *nod* genes through horizontal gene transfer (HGT) [23].

Rhizobia are often included among the so-called plant growth-promoting rhizobacteria (PGPR) [27] because of the desirable traits they impart in addition to their nitrogen-fixing ability [28]. These traits support the establishment and survival of plants in adverse conditions, thereby promoting plant productivity [29]. PGPR strains can be either endophytic (i.e., microbes living within a plant without causing disease such as in the case of rhizobial symbionts) or rhizospheric (i.e., microbes residing around the root region, which is often enriched with root exudates) and may provide either direct or indirect benefits to the plant [29,30]. Direct mechanisms include nitrogen fixation, the production of phytohormones such as indole-3-acetic acid (IAA), mineral solubilization, and the production of beneficial molecules such as siderophores (which scavenge the iron required for plant growth) [31]. Indirect mechanisms involve the production of lytic enzymes and antibiotics for the control of plant pathogens [32]. Another notable indirect mechanism involves the production of 1-aminocyclopropane-1-carboxylic acid (ACC) deaminase that assists in alleviating the effects of stress (e.g., drought, salinity, heat, cold, and exposure to heavy metals) on plant growth, as it limits the formation of ethylene produced in response to stress (i.e., ACC, the immediate precursor of ethylene is degraded by ACC deaminase to alpha-ketobutyrate and ammonia) [31,33]. These abilities have been witnessed among *Mesorhizobium* whereby *M. ciceri*, *M. loti*, *M. tianshanense*, *M. huakuii*, *M. mediterraneum*, *M. temperatum*, and *M. amorphae* are capable of producing IAA to varying levels [34], and solubilize phosphate [33,35,36]. They are, however, unable to produce siderophores [27]; although, certain strains produce siderophores when exposed to particular pesticides and fungicides [37,38]. Furthermore, among the *Mesorhizobium*, they have been found to harbor the ACC deaminase (*acdS*) gene [39,40,41,42]; although, this protein seems to be mostly expressed during nodulation initiation [33].

Chickpea is composed of two main varieties, desi and Kabuli. Desi is characterized by small dark brown-colored seeds, purple flowers, and sometimes purple stems while the Kabuli variety has large light-colored seeds and white flowers [43]. Desi is considered the more primitive of the two varieties with its growth populated in South and Southeast Asia, Ethiopia, Iran, and Mexico while Kabuli is mainly grown in South America, North Africa, West Asia, the Mediterranean, and South Europe [44,45]. Desi is winter-adapted, able to grow on residual moisture after the main crop in South Africa, and tends to blossom in the early spring as temperatures start rising [43,46]. Kabuli on the other hand is adapted to summer [46].

In the current study, we focused on desi chickpea, their rhizobia, and their associated plant growth-promoting abilities. This pulse is the second most important legume crop globally and is grown for its high nutritional content both for human and livestock consumption [46,47,48]. Although Africa accounts for less than 6% of the global production, various regions in Sub-Saharan Africa have the potential to become important chickpea cultivation areas [48]. However, a cost-effective and environmentally sustainable approach to accomplishing this would be dependent on the use and/or availability of suitable rhizobial symbionts [49]. This is because of chickpea’s high level of specificity for *Mesorhizobium* symbionts that are unlikely to occur in soils outside the native range of this legume species [50,51]. Therefore, this study aimed to investigate the nodulation ability of widely sourced *Mesorhizobium* strains and to evaluate their inherent ability to promote plant growth. The findings of this study would thus be valuable for identifying suitable *Mesorhizobium* microsymbiont(s) for testing under field conditions, and for future development of inoculants to cultivate this legume crop efficiently and sustainably.

## 2. Material and Methods

### 2.1. Chickpea-Associated Mesorhizobium Strains

This study included a total of 21 *Mesorhizobium* isolates (Table 1). Of these, twelve were previously isolated from chickpea root nodules and obtained from various culture collections globally, while *Mesorhizobium* sp. IC59 represented a commercial inoculant strain (Soygro, Potchefstroom, South Africa). The remaining eight strains were isolated as described by Howieson and Dilworth [52] from root nodules of chickpea plants that were grown in Mbombela (25°25′33.8″ S 30°58′15.4″ E), Mpumalanga Province, South Africa, in soils with no history of chickpea cultivation. Briefly, the nodules were surface sterilized by soaking in 70% ethanol for one minute, then transferred into 3% *w*/*v* sodium hypochlorite and soaked for three minutes. Thereafter, they were rinsed six times with sterile distilled water. The surface sterilized nodules were then crushed on yeast mannitol agar (YMA) plates and incubated at 28 °C for 5 days. This was followed by purification of the colonies through single-colony culturing on YMA plates.

All other isolates were routinely grown on YMA and incubated at 28 °C for five days [52]. To confirm that these bacteria represent members of *Mesorhizobium*, we sequenced a ca. 1400-base pair fragment of the gene (*rpoB*) encoding the β-subunit of RNA polymerase [53] and compared it to those of other bacteria in the GenBank [54] database of the National Centre for Biotechnology Information (NCBI; http//www.ncbi.nlm.nih.gov/, accessed on 3 June 2022) by making use of blastN [54,55]. 

### 2.2. Nodulation and Plant Growth

The symbiotic properties of all the strains considered in this study were investigated by completing a nodulation assay in a glasshouse pot experiment. This experiment involved six genotypes of desi-type chickpeas [64]. These included a commercial light variety that was obtained from a seed company (AGT Foods Africa, Krugersdorp, South Africa) and a dark variety sourced from a local shop (Limpopo, South Africa), as well as four improved genotypes (ICCV3110, ICCV3111, ICCV3203, and ICCV4105) developed by the International Crops Research Institute for the Semi-Arid Tropics (ICRISAT). 

Seeds were prepared for planting according to Sauer and Burroughs [65] by soaking in 95% (*v*/*v*) ethanol for 30 s, followed by soaking in 3.5% (*w/v*) sodium hypochlorite for five minutes. These surface sterilized seeds were rinsed six times with sterile distilled water and imbibed in sterile distilled water for 3 h. The seeds were then placed onto 15% (*w*/*v*) water agar (VWR International bvba Geldenaaksenbaan Leuven, Belgium) and incubated at 28 °C for two days to facilitate germination. 

The glasshouse experiment utilized a factorial experiment design that consisted of the six chickpea genotypes, 21 *Mesorhizobium* strains, a positive control (i.e., plants provided with a source of nitrogen), and a negative control (i.e., plants that did not receive nitrogen), in a completely randomized layout including three replicates. Sterile sand was used as the growth medium and the glasshouse temperature was maintained at 25 °C. Inoculants were prepared for each strain, by growing them for five days in yeast mannitol broth [52] at 28 °C. We then used 2 mL of this solution to inoculate two pregerminated seeds that had been planted in a 15 cm (1.9 L) pot. Nitrogen-free Hoagland growth solution [66] was applied thrice a week, while the positive control was provided with nitrogen in the form of 5 mL 0.05 M potassium nitrate per plant once a week. The experiment took place over 45 days after which the plants were harvested; although, height measurements were taken at a one-week interval from the fourth week after planting. 

To check for nodulation, the roots of the harvested plants were thoroughly rinsed with tap water whereafter the nodules were carefully excised from the roots. The nodules from a representative of each replicate were picked and cut through to ascertain whether the interior is pink (indicative that leghaemoglobin was formed to control oxygen levels in the nodule, ensuring a functional nitrogenase), thereby confirming the effectiveness of the nodules [52]. Nodule fresh weight (NFW) was recorded, and the nodules were stored in falcon tubes containing silica for further processing. Bacterial isolation was performed on the nodules of the chosen representative (three nodules per replicate) to confirm the identity of the bacteria. This was achieved by *rpoB* gene sequencing (as discussed above) followed by sequence comparison with the original sequences.

The shoots were dried in an air-circulating oven at 60 °C for 72 h after which shoot dry weight (SDW) was recorded. We then estimated relative symbiotic effectiveness (RSE) [67] using the formula $\frac{SDW\;\left(inoculated\;sample\right)}{SDW\;\left(CON+N\right)}\times 100$, where CON + N represents, positive control. Accordingly, RSE-values of <35% would indicate ineffective symbiosis (IE), values of 35–50% would indicate less effective (LE), while values of 51–80% and >80% would respectively indicate effective (E) and highly effective (HE) symbiosis.

### 2.3. Biochemical and Physiological Characterization

In this study, IAA production and phosphate solubilization were tested for all of the bacteria included. For the IAA assay, isolates were grown in tryptone yeast broth (TYB) and incubated at 28 °C for 5 days in a shaking incubator at 150 rpm. For each strain, 250 μL of this culture was then inoculated into 4 mL of broth consisting of 5 mM tryptophan (Merck, Midrand, South Africa) and mannitol (MonCon, Sandton, South Africa). This was followed by incubation at 28 °C for 5 days in a shaking incubator at 150 rpm. The bacterial cells were then removed by centrifugation at 3000 rcf for 30 min. Of the supernatant, 2 mL was mixed with 2 drops of orthophosphoric acid and 4 mL of Salkwoski’s reagent (1 mL 0.5 M FeCl_3,_ and 50 mL 70% perchloric acid (HClO_4_)). The mixture was then incubated at room temperature for 20–30 min [68,69]. 

A positive test for IAA production was indicated by a color change to red/purple (indolic compounds). Quantification was performed by calorimetrically measuring the absorbance at 530 nm with uninoculated YM broth used as negative control [68]. A 1000 µg/mL IAA stock solution was prepared by adding 10 mg IAA (SIGMA-ALDRICH Co., St. Louis, MO, USA) to 10 mL of acetone and swirled for the IAA to dissolve completely. A standard curve was constructed using pure IAA concentrations of 0 μg/mL, 5 μg/mL, 10 μg/mL, 20 μg/mL, 50 μg/mL, and 100 μg/mL with the software Origin (Pro) v8.5 (OriginLab Corporation, Northampton, MA, USA). The gradient of the standard curve was calculated from which the concentration of the test strains was determined using Beer Lambert’s law [70]: A = Ɛlc, where A = absorbance, Ɛ = absorptivity constant, l = cuvette length, and c = concentration. The resultant concentrations of the test strains were plotted on a bar graph with a 5% standard error on the data in Origin (Pro) v8.5.

To evaluate phosphate solubilization, single colonies were picked with a sterile inoculation loop from 5-day-old YMA cultures and spotted onto Pikovskaya’s medium [71]. Two colonies were spotted on the same plate for replication purposes. The plates were then incubated at 28 °C for 5 days after which the colonies were observed for phosphate solubilization. This was indicated by the formation of a clear zone or halo around the colony [29]. Phosphate solubilizing was estimated by taking into account the diameter of the colony (i.e., the diameter of the colony after incubation) and the halo zone diameter (i.e., two times the distance from the center of the colony to the end of the clear zone in a given direction). The phosphate-solubilizing index (PSI) was calculated as $\frac{Colony\;diameter+Halozone\;diameter}{Colony\;diameter}$ [29]. The obtained PSI of the test strains was plotted on a bar graph with a 5% standard error on the individual PSI values. 

### 2.4. Data Analysis

Two-way analysis of variance (ANOVA) using R (v.4.2.1) software [72] was used to assess the effect of the chickpea genotype and the *Mesorhizobium* strain on each other and their interactions with SDW, NFW, and plant heights. The Akaike information criterion (AIC) was used to select the best-fit model to explain variation in the dependent variables. Table of *p* values for all possible pairwise comparisons of least square means (lsmeans), and the letters (letter display) were conducted using Tukey’s honestly significant difference (Tukey’s HSD) post hoc test at a confidence level of 95% in R (v.4.2.1). Correlation analyses were conducted using R to test if NFW was correlated with SDW and average height. For visualization and presentation of the results generated, bar graphs were plotted using Origin (Pro) v8.5 software (OriginLab Corporation, Northampton, MA, USA). 

## 3. Results

### 3.1. Nodulation and Plant Growth

Nodulation was observed for most of the *Mesorhizobium* strains tested. The only exceptions were strains DSM30133, LMG17149, and SEMIA396, which failed to form nodules on any of the genotypes tested. In addition, strain P13.3 failed to nodulate the dark variety desi chickpea that was sourced from a local shop. As expected, there was no nodule formation on either the negative or the positive controls. 

Based on the ANOVA analysis (Table 2), both *Mesorhizobium* symbiont and chickpea genotypes affected the measured parameters (i.e., height, SDW, and NFW) significantly (*p* < 0.001). However, there were no interaction effects between the factors ‘strain’ and ‘genotype’ for all three parameters considered. This meant that the effect of symbiont on nodulation, height, SDW, and NFW was not significantly influenced by the chickpea genotype, and vice versa (i.e., the effect of chickpea genotype on nodulation, height, SDW, or NFW was not significantly influenced by the rhizobial strain used). 

**Figure 1:**
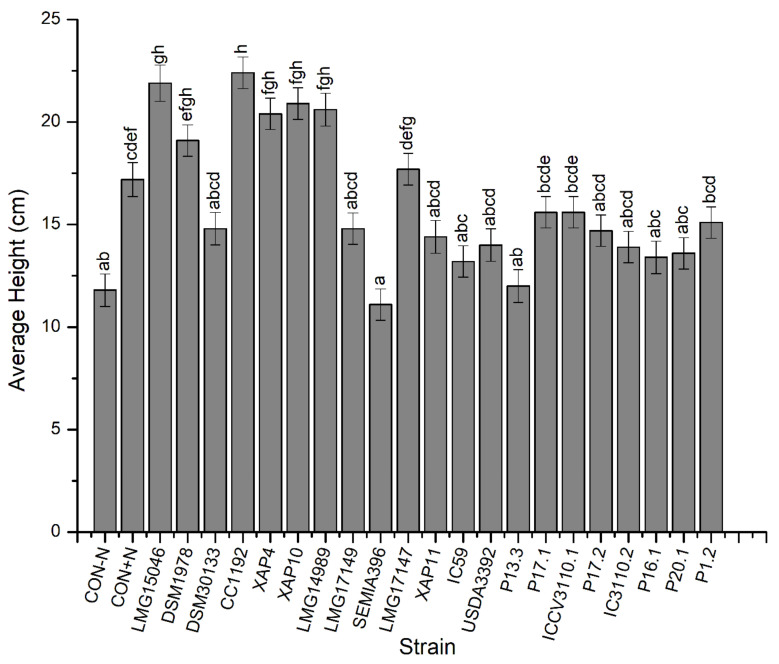
The effects of the *Mesorhizobium* isolates on the average height of chickpea plants. Bars are means ± standard errors, *n* = 3. Different letters (a–h) above the bars indicate treatments with significant differences (*p* < 0.05).

**Figure 2:**
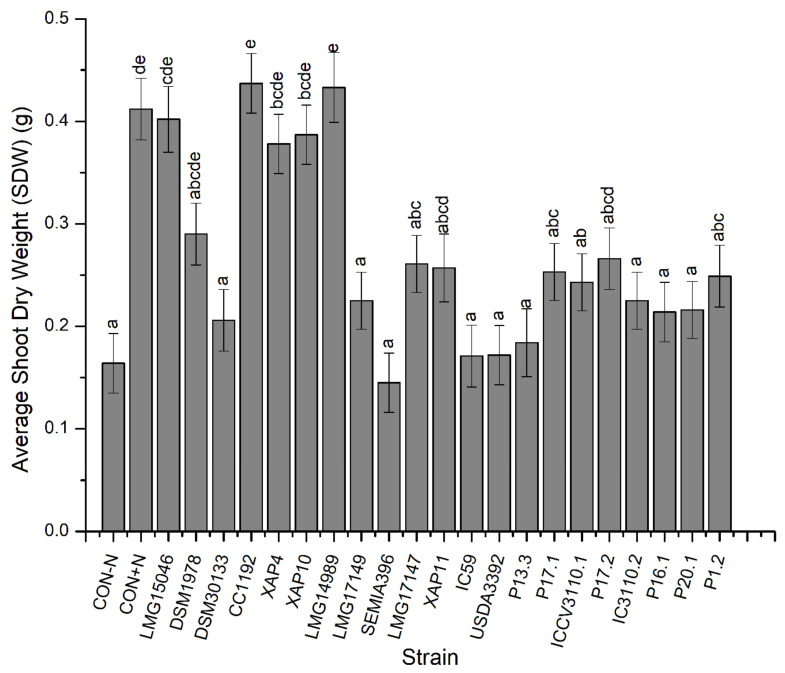
The effects of the *Mesorhizobium* isolate on shoot dry weight (SDW). Bars are means ± standard errors, *n* = 3. Different letters (a–e) above the bars indicate treatments with significant differences (*p* < 0.05).

**Figure 3:**
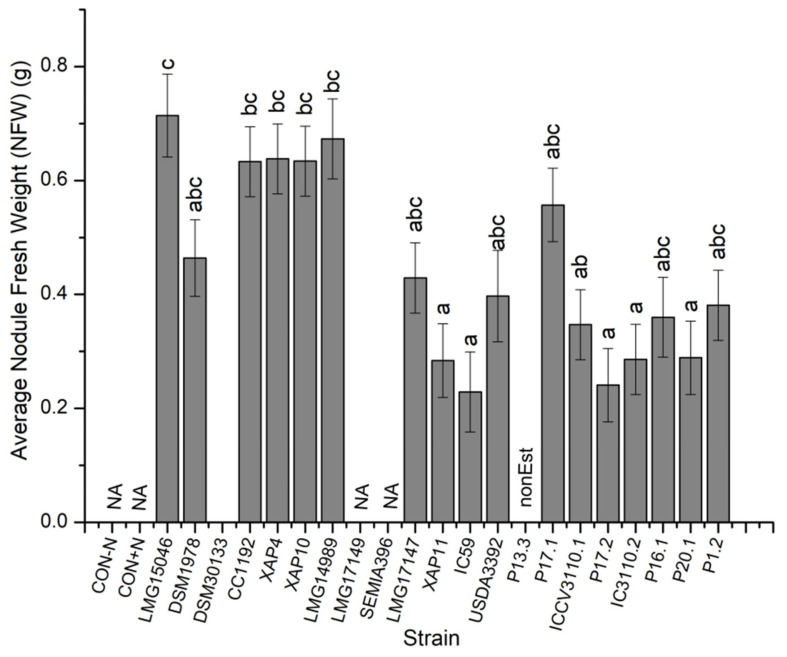
The graph of the effects of the *Mesorhizobium* isolate on nodule fresh weight (NFW) of chickpea. Bars are means ± standard errors, *n* = 3. Different letters (a–c) above the bars indicate treatments with significant differences (*p* < 0.05). NA = no nodules formed, nonEst = not estimated.

The results of the effect of *Mesorhizobium* strain on height, SDW, and NFW measurements of chickpeas are presented in Figure 1, Figure 2 and Figure 3, respectively. From these results, plants inoculated with strain CC1192 grew significantly higher when compared to plants inoculated with strains DSM30133, LMG17149, SEMIA396, LMG17147, XAP11, IC59, USDA3392, P13.3, P17.1, ICCV3110.1, P17.2, IC3110.2, P16.1, P20.1, or P1.2, and both the negative and positive controls (*p* < 0.05) (Figure 1). However, the height of plants inoculated with this strain was not significantly different from those of plants inoculated with strains LMG15046, DSM1978, XAP4, XAP10, or LMG14989 (Figure 1; *p* > 0.05).

For SDW, strains CC1192 and LMG14989 performed significantly better than strains DSM30133, LMG17149, SEMIA396, LMG17147, XAP11, IC59, USDA3392, P13.3, P17.1, ICCV3110.1, P17.2, IC3110.2, P16.1, P20.1, and the negative control (Figure 2) (*p* < 0.05). Measurements of SDW for plants inoculated with these two strains (i.e., CC1192 and LMG14989) were, however, not significantly different from those of plants inoculated with strains LMG15046, DSM1978, XAP10, XAP4, nor the positive control (*p* > 0.05) (Figure 2).

For NFW, plants inoculated with strain LMG15046 significantly outperformed those inoculated with strains XAP11, IC59, ICCV3110.1, P17.2, IC3110.2, and P20.1 (Figure 3; *p* < 0.05). It (LMG15046) was, however, not significantly different from those of plants inoculated with strains DSM1978, CC1192, XAP4, XAP10, LMG14989, LMG 17147, USDA3392, P17.1, P16.1, and P1.2 (Figure 3; *p* > 0.05). Interestingly, strain P13.3 showed poor nodulation for most of the chickpea genotypes used and was not considered in the NFW ANOVA analysis. Interestingly, NFW revealed strong significant positive correlations with SDW (r = 0.73; *p <* 0.001) and average height (r = 0.64; *p* < 0.001).

**Figure 4:**
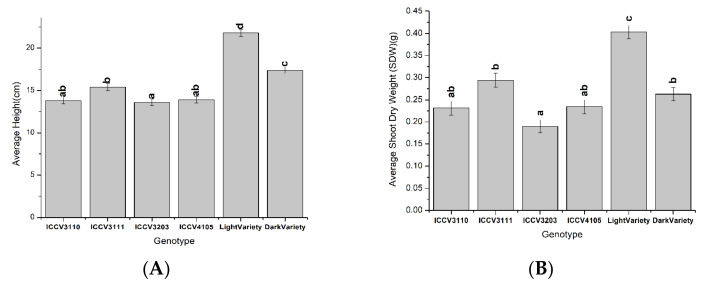

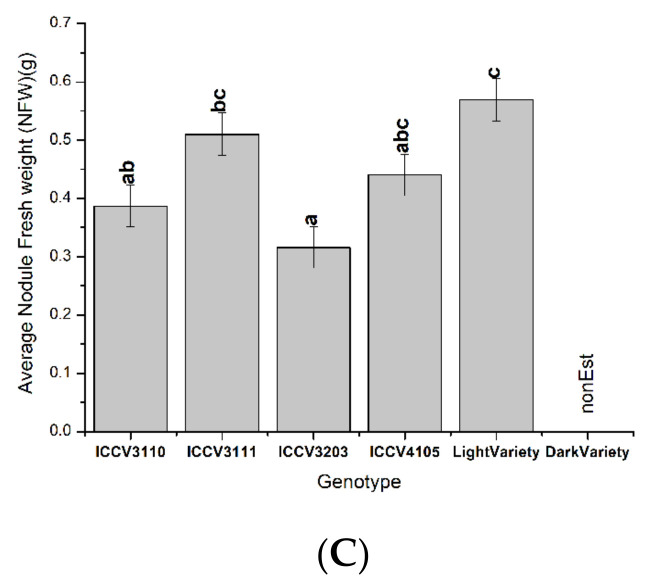
The effects of the factor chickpea genotype on average height (**A**), average shoot dry weight (SDW) (**B**), and average nodule fresh weight (NFW) (**C**). Bars are means ± standard errors, *n* = 3. Different letters (a–d) above the bars indicate treatments with significant differences (*p* < 0.05).

In terms of the effect of the chickpea genotype on the measurements recorded (Figure 4), the light variety generally showed higher performance than all the other genotypes, while the ICCV3203 genotype performed poorly. Among all genotypes, the light variety had significantly (*p* < 0.05) higher height and SDW values (Figure 4a,b), and the dark variety grew significantly (*p* < 0.05) higher than the four improved genotypes (Figure 4a). With regards to NFW (Figure 4c), the light variety and the ICCV3111 and ICCV4105 genotypes did not differ significantly (*p* > 0.05). The values for genotype ICCV3110 were also not significantly different from those of genotypes ICCV3111, ICCV3203, and ICCV4105 (*p* > 0.05). NFW values were not determined for the dark variety as it did not form nodules with strain P13.3.

Symbiotic effectiveness among the respective *Mesorhizobium* strains and chickpea genotypes varied widely (Table 3). Generally, the RSE for the light and dark variety genotypes ranged between 53.4% and 167.9%, indicating effective to highly effective symbiotic effectiveness for all the strains included in this study. Strain LMG15046 was highly effective (RSE values ranging from 88.0 to 156.1%) on all genotypes except ICCV4105 on which it yielded an RSE value of 75.2%. Strain CC1192 produced RSE values of 69.4–73.9% on genotypes ICCV3110 and ICCV3111, but were highly effective on the remaining genotypes, yielding RSE values of 91.1–163.2%. Strain XAP10 was LE with genotype ICCV3111, E with genotype ICCV3203, but HE for the rest of the genotypes considered. Strains XAP11, LMG17147, LMG17149, and USDA3392 were highly effective only on the dark and light variety chickpea genotypes. Strain SEMIA396 was generally ineffective and highly effective only on the dark variety. The six strains isolated from root nodules sourced from Mpumalanga mostly performed well only with the light and dark variety of chickpea, with only strain P17.2 also being highly effective (RSE value of 85.5%) on one of the improved genotypes (i.e., ICCV3110). 

### 3.2. Plant Growth Promotion Characteristics

The plant growth-promoting properties investigated in this study were IAA production and phosphate solubilization. Siderophore and ACC deaminase production were also evaluated using standard assays [73,74], but none of the 21 strains tested positive (results not shown). Nevertheless, all the strains, except the Mpumalanga root nodule isolates, were able to solubilize phosphate to varying degrees according to the PSI (Table 4). These values were interpreted according to Gupta and Pandey [29]. PSI-values of one indicated no phosphate solubilization (NPS), while PSI-values larger than one, but below two, were regarded as low levels of phosphate solubilization (LPS). PSI-values ≥ 2 indicated high levels of phosphate solubilization (HPS). Accordingly, all the strains that solubilized P were categorized as HPS, with the only exceptions being the LPS strains IC59, DSM1978, and LMG14989 (Table 4). 

The results of the IAA production indicated that all the strains considered were positive, but the amount produced varied across the strains tested (Table 4). Following Brigido et al. [34], the level of IAA production was denoted as low production (LP, <15 µgml^−1^), medium production (MP, 15–30 µgmL^−1^), high production (HP, 31–45 µgmL^−1^), and very high production (VHP, >45 µgmL^−1^). Accordingly, the commercial strain (IC59), produced the highest amount of IAA, while LMG17149 produced the lowest. Overall, one strain (LMG17149) was categorized as LP, one strain (XAP11) as MP, and two strains (LMG17147 and USDA3392) as HP. The rest of the strains, including the Mpumalanga isolates, were categorized as VHP (Table 4). 

## 4. Discussion

The findings of this study showed that nodulation efficiency varied widely depending on the *Mesorhizobium* strain and chickpea genotype used. For example, several strains such as LMG15046, CC1192, XAP4, XAP10, and LMG14989 produced higher nodule weights while the least nodule weights were observed for XAP11, IC59, and most of the field-isolated strains in this study. Higher nodule weights were further observed for the light variety compared to ICCV3203 and ICCV3110 genotypes of chickpea. This is consistent with previous reports such as those conducted on faba bean in Ethiopia [75] and chickpea in Tunisia and Ethiopia [76,77], where inoculation with different strains yielded varying nodule numbers and weights. Moreover, varying effects of the *Mesorhizobium* strains on the shoot dry weights, average heights, and symbiotic efficiencies of different chickpea genotypes were observed. For example, strains LMG15046, CC1192, XAP4, XAP10, and LMG14989 consistently revealed high average heights, shoot dry weights, and symbiotic effectiveness over the rest of the strains including the South African field isolates and the negative control. Interestingly, these findings were also supported by strong significant positive correlations of NFW with SDW and average heights. Variations in shoot dry weights and symbiotic effectiveness with regard to chickpea genotypes were also observed in Tunisia and Ethiopia [67,77]. On the other hand, there were no significant interaction effects between the two factors ‘strains’ and ‘chickpea genotype’ observed in this study, which is consistent with other reports conducted in the field [77] or pot experiments [76]. These findings provide an array of effective strains, irrespective of the chickpea genotype, which could be tested in South African field conditions for chickpea production. 

The light and the dark variety desi chickpea genotypes performed better across the tested genotypes. Although the exact genotype of the dark variety is unknown, the light variety was sourced from AGT Foods Africa, which is a company that specializes in seed improvement. The better performance of the light variety likely reflects their aim for improving the yield and quality of their product [78]. However, for producing improved legume crops, including chickpea, breeding efforts typically focus on the plant’s ability to overcome abiotic stress such as drought, or biotic stress such as pathogens [79,80], with improvements in its symbiotic interactions rarely taken into consideration. For chickpea, this is important because the plant genotype–rhizobial symbionts interaction can be exploited to produce seeds that would be highly productive in particular soils [81]. Due to the substantial levels of heritability among chickpea genotypes for traits such as nodulation and seed yield, selection for these traits during breeding could affect permanent improvements to the crop [81]. 

An interesting finding from the current study was that three of the strains (namely, DSM30133, LMG17149, and SEMIA396) used did not nodulate any of the chickpea genotypes tested. Although these bacteria were originally isolated from chickpea nodules [56,57,58,59,60,61], the nodulation ability of only DSM30133 and SEMIA396 has been confirmed. A study from Brazil showed that SEMIA396 can nodulate chickpea effectively [82], while Crow et al. [83] confirmed this for DSM30133 (also known as strain 3HOa1). The latter strain apparently also forms ineffective nodules on legumes such as *Carmichaelia angustata*, *Clianthus puniceus* (G.Don) Sol. ex Lindl., and *Medicago sativa* L. [83]. Not much is known about the nodulation ability of strain LMG17149 (also known as strain 522), but Cadahía et al. [56] used Southern hybridization to show that it harbors nitrogen fixation genes typically encoded by rhizobia. In *Mesorhizobium*, genes determining the nitrogen-fixing symbiosis are carried in so-called symbiosis islands, which occur within chromosomally located integrative and conjugative elements (accordingly referred to as ICESyms) [59,84,85,86]. These elements are often implicated in HGT, including within and among chickpea-associated *Mesorhizobium* species and lineages [59,87,88]. The apparent inability of strains DSM30133, LMG17149, and SEMIA396 could thus point toward a type of dysregulation or inactivation of their symbiosis genes and/or the loss of entire ICESyms or components thereof [89,90]. Our future research will seek to resolve these issues using genomics approaches. 

All eight of the *Mesorhizobium* strains isolated from nodules that were obtained from field-cultivated chickpea in Mpumalanga were capable of nodulation, albeit mostly less effective than those obtained from elsewhere in the world. This is consistent with what was observed in an Australian study of the *Mesorhizobium* symbionts of chickpea in fields with no history of inoculation [91]. A large proportion of these naturalized strains displayed suboptimal compatibility with the plant genotypes tested, which in turn negatively impacted the nitrogen-fixing symbiosis [59]. This effect is particularly pronounced in non-native conditions, where legume–rhizobium compatibility strongly influences symbiotic efficiency [92]. In the case of chickpea *Mesorhizobium,* such inadequacies in naturalized strains have been ascribed to suboptimal interactions between chromosomally expressed genes/gene products and those expressed from horizontally acquired ICESyms [59]. It would be interesting to see whether this was also the case for the eight Mpumalanga strains used in the current study. 

Wide variation was observed among the rhizobia regarding the two-plant growth-promoting traits assayed, but these generally did not correspond with symbiotic effectiveness. As expected for *Mesorhizobium*, most of the strains produced high amounts of IAA [34,69], but some of the strains showed only limited phosphate solubilization and none of the Mpumalanga strains exhibited this trait. However, four of the strains (LMG15046, CC1192, XAP10, and XAP4) performed well across these two parameters (IAA and P solubilization) and also displayed high symbiotic effectiveness on at least four of the six chickpea genotypes tested. The isolates in this study could produce IAA and solubilize phosphate (except the South African isolates) but their consistency among the mentioned five well-performing strains was not observed. IAA producers are expected to facilitate a dense rooting system and consequently increased nodule formation [31]. Under field conditions, the ability to solubilize phosphate would be a desirable trait for most soils in Sub-Saharan Africa [93,94], as the use of these or strains with similar properties would boost the uptake of this nutrient by the plant [95,96]. Therefore, the five superior *Mesorhizobium* strains identified in the current study represent an important resource for the future development of suitable inoculants for agricultural use [97,98].

The commercial inoculant strain (*Mesorhizobium* sp. IC59) performed comparatively poorly in our study. Rhizobial inoculants are typically selected based on their plant growth-promoting capacity and efficacy in nitrogen fixation [52,98]. Although the inoculant strain excelled in terms of IAA production, chickpea plants inoculated with it did not grow significantly taller or produce significantly more biomass than our no-nitrogen control treatments. In terms of relative symbiotic effectiveness, it also lagged behind most of the strains sourced from culture collections and did not perform significantly better than any of the Mpumalanga strains. These findings thus highlight the need for effective inoculants, especially in Sub-Saharan Africa [48]. In other parts of Africa where chickpea has a long history of production [99,100,101,102], some including its native range [50,51], inoculant use is less crucial as suitable rhizobia might be present in the soil. In Sub-Saharan Africa, however, chickpea cultivation necessitates the use of effective nodulants, as has been undertaken in Australia, for example, with the use of strain CC1192 (included among the strains evaluated in the current study), which is widely used as chickpea inoculant in Australia [59].

## 5. Conclusions

In conclusion, this study screened several chickpea-associated *Mesorhizobium* strains for their ability to effectively interact with and promote the growth of several genotypes in a controlled environment in South Africa. From our findings, the LMG15046, CC1192, XAP4, XAP10, and LMG14989 strains, irrespective of chickpea genotype used, performed better than the remainder of the *Mesorhizobium* strains with regards to the parameters determined such as average height, shoot dry weight, and nodules fresh weight. To the best of our knowledge, this study is the first to also report the isolation of chickpea-associated *Mesorhizobium* strains from an uninoculated field in South Africa. It is, however, worth noting that these field strains showed less effectiveness compared to the globally sourced strains in terms of chickpea growth promotion in controlled conditions. We were also able to show that from the different chickpea genotypes considered, the light variety sourced from a local seed company outperformed the rest in terms of all the parameters tested including symbiotic effectiveness, under controlled conditions. As this study was conducted in such a controlled setting, we recommend expanding the research to include testing these five strains in a field setting to incorporate environmental conditions and other biotic factors. 

## Figures and Tables

**Table 1:** Chickpea-associated *Mesorhizobium* strains incorporated in this study, their source, and country of initial isolation. The respective genotypes of chickpea from which field nodules were collected for isolation of rhizobia in Mpumalanga South Africa are indicated in brackets.

Strain ^1^	Original Host ^2^	Region of Original Isolation	References
*Mesorhizobium ciceri* LMG14989^T^	*Cicer arietinum* L.	Spain	[56,57,58]
*Mesorhizobium ciceri* CC1192	*Cicer arietinum* L.	Israel	[59]
*Mesorhizobium mediterranean* USDA3392	*Cicer arietinum* L.	Brazil	[58,60]
*Mesorhizobium* sp. SEMIA396	*Cicer arietinum* L.	North America	[61]
*Mesorhizobium* sp. DSM30133	*Cicer arietinum* L.	Belgium	[56]
*Mesorhizobium* sp. DSM1978	*Cicer arietinum* L.	India	[62]
*Mesorhizobium* sp. XAP4	*Cicer arietinum* L.	USA (Wisconsin)	Unknown
*Mesorhizobium* sp. XAP10	*Cicer arietinum* L.	Australia	Unknown
*Mesorhizobium* sp. XAP11	*Cicer arietinum* L.	India	Unknown
*Mesorhizobium* sp. LMG15046	*Cicer arietinum* L.	India	[56]
*Mesorhizobium* sp. LMG17147	*Cicer arietinum* L.	India	[56]
*Mesorhizobium* sp. LMG17149	*Cicer arietinum* L.	Russia	[56]
*Mesorhizobium* sp. IC59 *	*Cicer arietinum* L.	India	[63]
*Mesorhizobium* sp. ICCV3110.1	*Cicer arietinum* L. (ICCV3110)	Mbombela, Mpumalanga, South Africa	This study
*Mesorhizobium* sp. P16.1	*Cicer arietinum* L. (ICCV3110)	Mbombela, Mpumalanga, South Africa	This study
*Mesorhizobium* sp. P13.3	*Cicer arietinum* L. (ICCV92944)	Mbombela, Mpumalanga, South Africa	This study
*Mesorhizobium* sp. P17.1	*Cicer arietinum* L. (ICCV4105)	Mbombela, Mpumalanga, South Africa	This study
*Mesorhizobium* sp. P17.2	*Cicer arietinum* L. (ICCV4105)	Mbombela, Mpumalanga, South Africa	This study
*Mesorhizobium* sp. P20.1	*Cicer arietinum* L. (ICCV4110)	Mbombela, Mpumalanga, South Africa	This study
*Mesorhizobium* sp. P1.2	*Cicer arietinum* L. (ICCV4110)	Mbombela, Mpumalanga, South Africa	This study
*Mesorhizobium* sp. IC3110.2	*Cicer arietinum* L. (ICCV3110)	Mbombela, Mpumalanga, South Africa	This study

^1^ Strain code (where applicable): LMG—BCCM/LMG, Belgium, CC1192—Centre for Rhizobium Studies, Murdoch University, Australia, USDA—IL, USA, DSM—DSMZ, Germany, XAP—SARCC, South Africa, and SEMIA—Rhizobium Culture Collection, Porto Alegre, Brazil. ^2^ ICCV—Indian Chickpea Cultivar, ICRISAT. * Commercial inoculant (Soygro, Potchefstroom, South Africa).

**Table 2:** Analysis of Variance (ANOVA) results of the effect of the *Mesorhizobium* strains and chickpea genotype on the height, shoot dry weight (SDW), and nodule fresh weight (NFW) of inoculated plants.

Measured Parameters	Sources of Variations		
	Strain	Genotype	Genotype × Strain
Height	*p* < 0.001	*p* < 0.001	*p* > 0.05
SDW	*p* < 0.001	*p* < 0.001	*p* > 0.05
NFW	*p* < 0.001	*p* < 0.001	*p* > 0.05

**Table 3:** Relative symbiotic effectiveness (RSE) scores of the *Mesorhizobium* strains on the six desi chickpea genotypes studied.

	Light Variety			Dark Variety			ICCV3110			ICCV3111			ICCV3203			ICCV4105		
Strain	SDW	RSE	Score	SDW	RSE	Score	SDW	RSE	Score	SDW	RSE	Score	SDW	RSE	Score	SDW	RSE	Score
CON− N	0.28	66.3	IE	0.18	70.4	E	0.12	27.6	IE	0.17	27.5	IE	0.07	19.2	IE	0.16	42.3	LE
CON+ N	0.43	100.0	HE	0.25	100.0	HE	0.44	100.0	HE	0.63	100.0	HE	0.35	100.0	HE	0.39	100.0	HE
P16.1	0.40	94.1	HE	0.34	132.4	HE	0.13	28.7	IE	0.13	21.3	IE	0.18	50.9	E	0.11	28.5	IE
P13.3	0.34	80.3	HE	0.22	87.0	HE	0.15	34.0	IE	0.17	26.9	IE	0.10	29.3	IE	0.13	33.3	IE
P17.1	0.38	88.5	HE	0.37	145.1	HE	0.20	46.0	LE	0.27	43.7	LE	0.16	44.7	LE	0.14	37.6	LE
P17.2	0.41	96.0	HE	0.15	58.5	E	0.37	85.5	HE	0.25	39.5	LE	0.26	75.3	E	0.16	41.9	LE
P20.1	0.27	62.5	E	0.19	76.3	E	0.21	47.6	LE	0.17	26.9	IE	0.22	61.9	E	0.25	63.9	E
P1.2	0.33	76.6	E	0.30	118.6	HE	0.13	29.2	IE	0.29	46.7	LE	0.18	50.9	E	0.27	69.1	E
IC3110.2	0.30	70.3	E	0.21	81.8	HE	0.29	66.2	E	0.24	37.6	LE	0.13	36.0	LE	0.19	50.1	E
ICCV3110.1	0.34	78.9	E	0.28	109.5	HE	0.17	37.9	LE	0.35	55.2	E	0.16	46.5	LE	0.17	45.3	LE
IC59	0.29	67.4	E	0.14	56.5	E	0.18	41.4	LE	0.13	20.0	IE	0.13	38.4	LE	0.16	41.9	LE
LMG17147	0.40	94.1	HE	0.22	86.2	HE	0.24	54.7	E	0.25	40.5	LE	0.19	54.7	E	0.26	68.7	E
XAP11	0.37	87.1	HE	0.32	125.3	HE	0.16	36.8	LE	0.30	48.0	LE	0.16	46.1	LE	0.24	62.6	E
SEMIA396	0.23	53.4	E	0.22	85.0	HE	0.07	14.9	IE	0.18	28.8	IE	0.08	24.0	IE	0.10	25.5	IE
LMG17149	0.35	80.8	HE	0.31	121.7	HE	0.20	46.7	LE	0.14	22.4	IE	0.18	52.8	E	0.17	44.5	LE
DSM1978	0.43	101.2	HE	0.29	115.4	HE	0.16	35.6	LE	0.45	72.3	E	0.20	57.6	E	0.20	53.1	E
DSM30133	0.27	62.1	E	0.20	78.3	E	0.23	51.7	E	0.27	42.4	LE	0.15	42.7	LE	0.14	35.9	LE
USDA3392	0.31	73.1	E	0.17	68.4	E	0.08	18.4	IE	0.19	29.9	IE	0.12	34.3	IE	0.15	39.7	LE
LMG15046	0.48	112.9	HE	0.40	156.1	HE	0.38	88.0	HE	0.56	88.8	HE	0.31	89.2	HE	0.29	75.2	E
XAP4	0.72	167.9	HE	0.32	126.5	HE	0.28	63.9	E	0.40	64.5	E	0.28	79.6	E	0.28	72.2	E
CC1192	0.69	161.6	HE	0.41	163.2	HE	0.30	69.4	E	0.46	73.9	E	0.32	91.1	HE	0.44	114.5	HE
LMG14989	0.66	154.6	HE	0.27	107.1	HE	0.42	96.6	HE	0.49	77.9	E	0.24	69.1	E	0.51	132.2	HE
XAP10	0.61	142.2	HE	0.30	118.6	HE	0.44	101.6	HE	0.31	49.1	LE	0.21	60.5	E	0.45	117.9	HE

CON − N = negative control, CON + N = positive control, HE = highly effective, E = effective, LE = low level of effectiveness, IE = ineffective.

**Table 4:** Production and scoring of IAA production and phosphate solubilization by different *Mesorhizobium* isolates used in this study.

Strains	Indole Acetic Acid (IAA)		Phosphate Solubilization Index (PSI)	
	Concentration (µg/mL)	Score	PSI	Score
P16.1	108	VHP	ns	NPS
P13.3	105	VHP	ns	NPS
P17.1	92	VHP	ns	NPS
P17.2	97	VHP	ns	NPS
P20.1	104	VHP	ns	NPS
P1.2	96	VHP	ns	NPS
IC3110.2	103	VHP	ns	NPS
ICCV3110.1	105	VHP	ns	NPS
IC59	163	VHP	1.3	LPS
LMG17147	38	HP	2.4	HPS
XAP11	27	MP	2.3	HPS
SEMIA396	99	VHP	2.7	HPS
LMG17149	10	LP	3.3	HPS
DSM1978	48	VHP	1.7	LPS
DSM30133	46	VHP	2.4	HPS
USDA3392	31	HP	2.4	HPS
LMG15046	73	VHP	2.2	HPS
XAP4	105	VHP	3.5	HPS
CC1192	47	VHP	2.6	HPS
LMG14989	78	VHP	1.4	LPS
XAP10	94	VHP	2.3	HPS

ns = no solubilization, LP = low production, MP = medium production, HP = high production, VHP = very high production, NPS = no phosphate solubilization, LPS = low phosphate solubilization, HPS = high phosphate solubilization.

## Data Availability

The data supporting the reported results in this article is presented in form of Figures and Tables in this article, hence there is no extra data elsewhere.
